# Admission D-dimer to lymphocyte counts ratio as a novel biomarker for predicting the in-hospital mortality in patients with acute aortic dissection

**DOI:** 10.1186/s12872-023-03098-x

**Published:** 2023-02-05

**Authors:** Yansong Xu, Silei Liang, Zheng Liang, Cuiqing Huang, Yihuan Luo, Guanbiao Liang, Wei Wang

**Affiliations:** 1 Emergency Surgery Department, The First Affiliated Hospital of Guangxi Medical University, Nanning, China; 2 Trauma Center, The First Affiliated Hospital of Guangxi Medical University, Nanning, China; 3 Medical Department, The First Affiliated Hospital of Guangxi Medical University, Nanning, China; 4 Cardiothoracic Surgery Department, The First Affiliated Hospital of Guangxi Medical University, Nanning, China

**Keywords:** Acute aortic dissection, D-dimer, D-dimer to lymphocyte, Count ratio, In-hospital mortality

## Abstract

**Background:**

Inflammatory factors are well-established indicators for vascular disease, but the D-dimer to lymphocyte count ratio (DLR) is not measured in routine clinical care. Screening of DLR in individuals may identify individuals at in-hopital mortality of acute aortic dissection (AD).

**Methods:**

A retrospective analysis of clinical data from 2013 to 2020 was conducted to identify which factors were related to in-hospital mortality risk of AD. Baseline clinical features, cardiovascular risk factors, and laboratory parameters were obtained from the hospital database. The end point was in-hospital mortality. Forward conditional logistic regression was performed to identify independent risk factors for AA in-hospital death. The cutoff value of the DLR should be ideally calculated by receiver operating characteristic (ROC) analysis.

**Results:**

The in-hospital mortality rate was 15% (48 of 320 patients). Patients with in-hospital mortality had a higher admission mean DLR level than the alive group (1740 vs. 1010, *P* < .05). The cutoff point of DLR was 907. The in-hospital mortality rate in the high-level DLR group was significantly higher than that in the low-level DLR group (*P* < .05). Univariate analysis showed that 8 of 38 factors were associated with in-hospital mortality (*P* < .05), including admission WBC, neutrophils, lymphocytes, neutrophils/lymphocytes (NLR), prothrombin time (PT), heart rate (HR), D-dimer, and DLR. In multivariate analysis, DLR (odds ratio [OR] 2.127, 95% CI 1.034–4.373, *P* = 0.040), HR (odds ratio [OR] 1.016, 95% CI 1.002–1.030, *P* = 0.029) and PT (odds ratio [OR] 1.231, 95% CI 1.018–1.189, *P* = 0.032) were determined to be independent predictors of in-hospital mortality (*P* < .05).

**Conclusion:**

Compared with the common clinical parameters PT and HR, serum DLR level on admission is an uncommon but independent parameter that can be used to assess in-hospital mortality in patients with acute AD.

## Introduction

Acute aortic dissection (AAD) is an infrequent but life-threatening condition. Surgery is the most effective treatment for AAD. Due to the properties of rapid onset, quick progression and mortality, in-hospital mortality rates are still between 18 and 30% after surgery [1, 2]. Greater insight into the prognosis of AAD is also needed for optimization of treatment strategies [3]. We believe that the early prognosis can be determined from the basic characteristics and symptoms of patients.

The pathogenesis of aortic dissection is very complex and includes hereditary diseases and aortic wall pathological disease. Recent studies have found that the immune inflammatory mechanism is closely related to the occurrence of aortic dissection, arterial media degeneration, and vascular remodeling. In the tissues of AD patients, a large number of inflammatory cells were detected in the media and adventitia of vessels. Research found that T lymphocyte activation in patients with aortic dissection resulted in a decrease in the number and ratio of peripheral blood lymphocytes. Acute dissection patients with a strong systemic inflammatory response have a worse prognosis. This finding is consistent with the finding that D dimer levels in the peripheral blood of AD patients are significantly increased. Inflammatory factors and D-dimer are related to the prognosis of aortic dissection. The prognosis of aortic dissection is a complex process involving multiple factors. However, there is no study on the relationship between DLR and aortic dissection.

D-dimer, a kind of degradation product of cross-linked fibrin, indicating fibrinolytic activities in AD, could be detected in peripheral blood within 10 min [4]. The utility of D-dimer assessment in the diagnosis and prognosis of acute aortic disease, including AAD, is well recognized [3, 5–7]. The European Society of Cardiology Guidelines recommends admission D-dimer elevation as a biomarker for a diagnostic workup of AAD [8], and previously reported studies have shown a correlation between D-dimer concentrations and the prognosis of AAD [3, 9, 10]. Inflammatory factors are heritable risk factors for vascular disease, but their role in the prognosis of AAD is unclear. The majority of lymphocytes in peripheral blood are in the resting state. However, tissue injury may affect peripheral blood composition. Several animal models have shown either lymphocyte loss or lymphocyte dysfunction following various models of vessel injury [11–13]. Clinical research has shown that there is a significant decrease in T lymphocytes in the peripheral blood of patients with AAD [14]. Previous studies described that lymphopenia was associated with poor prognosis in critical patients, including AAD [13, 15, 16]. Unlike NLR and PLR, the application value is widely recognized in the prognosis of AAD [16–18]. There are few studies on the relationship between DLR and the prognosis of patients with AAD.

We conducted a retrospective study in which we investigated admission DLR in relation to early outcome among these patients to determine whether admission DLR can be used as an early indicator of in-hospital mortality in patients with AAD.

## Methods

### Selection of participants

This single-center, retrospective study comprised consecutive patients with AAD who were admitted to the emergency department, vascular surgery department, cardiac surgery department and critical care center of the First Affiliated Hospital of Guangxi Medical University between 2013 and 2020. Patients with cardiac arrest on arrival, presentation later than 48 h after the onset of AAD, operative history of AD, traumatic arterial dissection, and unavailability of D-dimer or/and lymphocyte values at the time of AAD diagnosis were excluded. A definite diagnosis of AD was made using contrast-enhanced computed tomography (CECT). or magnetic resonance imaging (MRI). AD was classified according to the Stanford classification. Patients with AD were divided into two groups: in-hospital mortality and alive groups.

### Data extraction and variable screening

We retrospectively reviewed the medical records of the patients according to ID from our electronic database by two doctors (Yansong Xu, Zheng Liang). Based on the current literature [19, 20], we selected the following variables: baseline characteristics, clinical features, laboratory results, imaging procedures, and patient outcomes. The baseline DLR was measured by dividing the D-dimer level by the lymphocyte count.

Baseline characteristics included sex, age, body mass index (BMI), and medical history, including diabetes mellitus, hypertension, drinking status, and smoking status. Clinical features included HR and systolic/diastolic blood pressure at admission. This study draws on peripheral blood leukocytes, hemoglobin, platelets, neutrophils, lymphocytes, plasma prothrombin time, fibrinogen, partial prothrombin time, serum total cholesterol, triglycerides, high-density lipoprotein cholesterol (hdl-c), low-density lipoprotein cholesterol (hdl-c), serum creatinine, blood urea nitrogen, brain natriuretic peptide, creatine kinase, creatine kinase isoenzyme, lactate dehydrogenase, and D-dimer.

### Statistical analysis

Student’s t test or the Mann‒Whitney U test was used for analysis in the case of normally or nonnormally distributed continuous variables. Mean and standard deviation (SD) were used for normally distributed variables: median and interquartile ranges (IQRs). Normal distributions are reported as the mean, non-normal distributions are reported as the median. Thechi-square test or Fisher’s exact test was used to compare the categorical variables between the two groups. The optimal cutoff values of DLR were determined using ROC. Multivariable analysis was performed for in-hospital mortality, including variables with a p value ofless than 0.05 in univariate analysis. Analyses were performed with SPSS 26.0, and a *p* value < 0.05 was considered statistically significant.

## Results

### Baseline characteristics

**Fig. 1:**
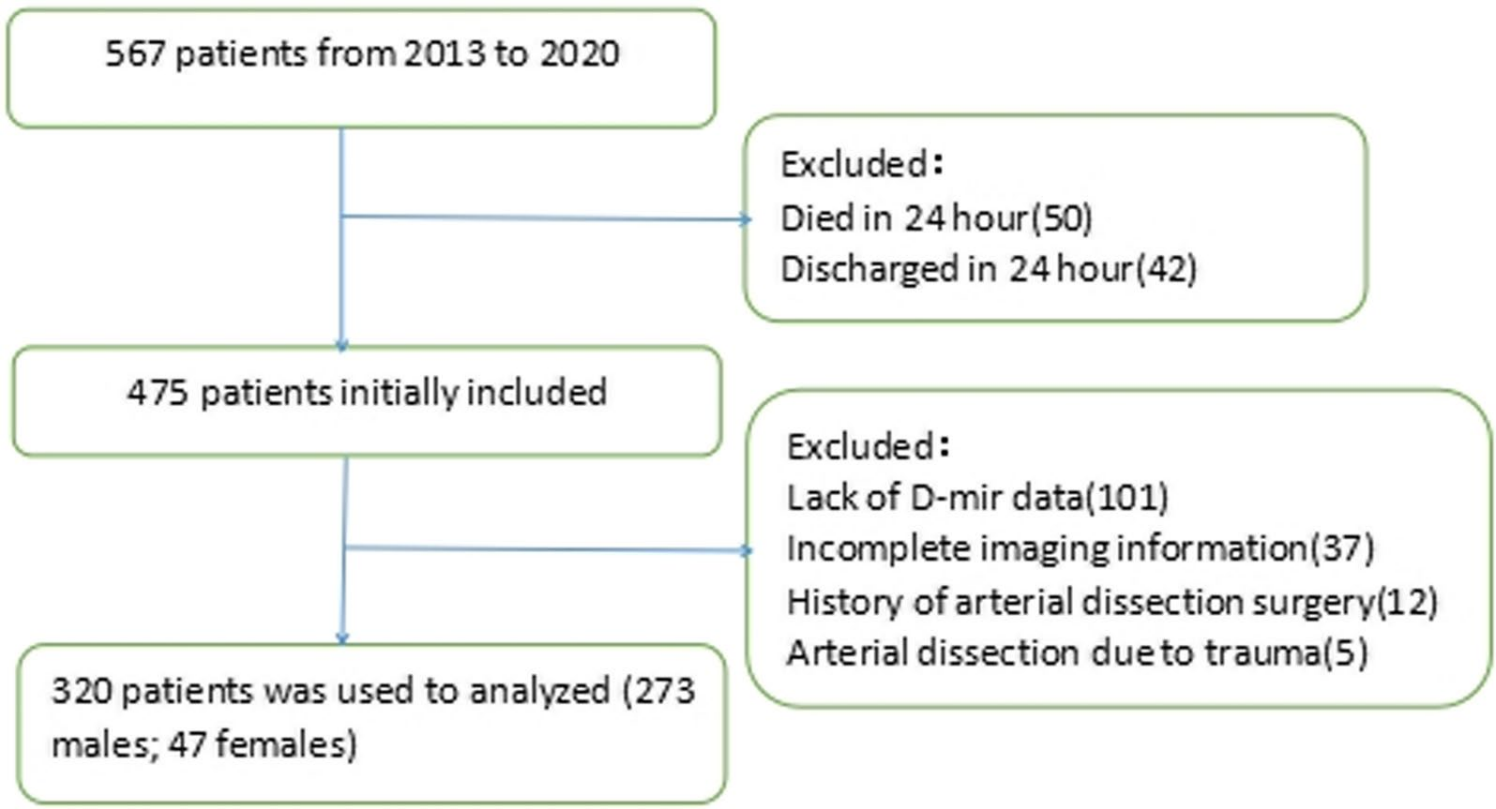
Screening flow chart

A total of 567 patients with AAD were admitted to our hospital between 2013 and 2020. A total of 247 patients with incomplete data were excluded. Finally, 320 patients with admission complete parameters were investigated, including 273 males and 47 females (Fig. 1). The relationships between different DLR groups and parameters are presentedin Table 1. Admission WBC, neutrophils, lymphocytes, NLR, PT, CK, D-dimer, DLR, and HR were significantly different (all *P* < 0.05) between the two groups.

**Table 1:** General characteristics of the study population

Variables		Outcome		F/t	*P*
		Alive	Mortality		
Sex	Male/Female	228/44	45/3	2.465	0.116
Age, mean (SD)	51.8(11.4)	52.3(11.5)	49.6(10.5)	0.115	0.156
Dissection type	A/B	85/187	18/30	0.730	0.393
Hypertension	No/Yes	84/16	188/32	0.114	0.736
Diabetes	No/Yes	260/12	48/48	1.148	0.284
Smoking	No/Yes	134/138	28/20	1.342	0.247
Drinking	No/Yes	142/130	28/20	0.615	0.433
WBC, mean (SD)	11(3.9)	10.7(3.8)	12.3(4)	0.124	**0.013**
HGB, mean (SD)	120.4(20.4)	121(20)	117(21)	0.085	0.216
PLT, mean (SD)	221.6(97.7)	221.4(93.3)	222.8(122.5)		
NEUT, mean (SD)	8.5(3.8)	8.2(3.7)	9.9(4)	0.001	**0.004**
LYM, mean (SD)	1.3(0.5)	1.3(0.5)	1.1(0.3)	4.959	**0.008**
PLR, md (IQR)	161.4(120.5–238.7)	159.2(120.5–236.4)	184.4(120.4–253.2)	0.611	0.357
NLR, md (IQR)	6.9(3.9–10.6)	6.9(3.7–10.1)	9.2(5.7–12.8)	0.071	**0.017**
PT, mean (SD)	12(1.6)	11.8(1.3)	12.7(2.4)	8.224	**0.018**
FIB, mean (SD)	4.8(1.7)	4.8(1.6)	4.6(2)	2.538	0.510
APTT, mean (SD)	31.6(5.0)	31.6(4.7)	32(6.4)	0.069	0.534
TC, mean (SD)	4.4(1.1)	4.4(1.1)	4.3(1.2)	3.335	0.517
TG, mean (SD)	1.2(0.9)	1.4(0.9)	1.3(0.9)	0.249	0.515
HDL, mean (SD)	1.1(0.3)	1.0(0.3)	1.0(0.3)	0.533	0.559
LDL, mean (SD)	2.6(0.9)	2.6(0.9)	2.5(0.9)	0.210	0.684
CR, md (IQR)	94(70.0–12.06)	134.7(68–187)	111(75–195.5)	3.208	0.091
UA, md (IQR)	382.4(170.8)	375(162)	433(211)	3.219	0.074
BNP, md (IQR)	534.8(155.7–1341.2)	497(139–1141)	743.4(311–2240)	6.339	0.145
CK, md (IQR)	84(52.0–185.7)	82.5(54–169.7)	95.5(43–309)	14.962	0.368
CKMB, md (IQR)	13(10.0–18.0)	13(10–17)	16(11.2–22.8)	5.780	0.313
LDH, md (IQR)	243(199.0–300.0)	240.2(197.2–291.7)	268.5(212–395)	4.553	0.064
HR, mean (SD)	88(20)	86(20	96(21)	0.500	**0.003**
SBP, mean (SD)	144(31)	144(30)	140(33)	0.383	0.385
DBP, mean (SD)	85(19)	85(19)	83(19)	0.066	0.470
D-dmir, md (IQR)	1186(664–2818)	1136(650–2767	1786(924–6608)	20.857	**0.001**
BMI, mean (SD)	23(3.9)	23(3.8)	22(4.3)	2.370	0.267
DLR	<907/ ≥ 907	128/144	12/36	8.067	**0.005**

Bold value indicates *P* value < 0.05

**Fig. 2:**
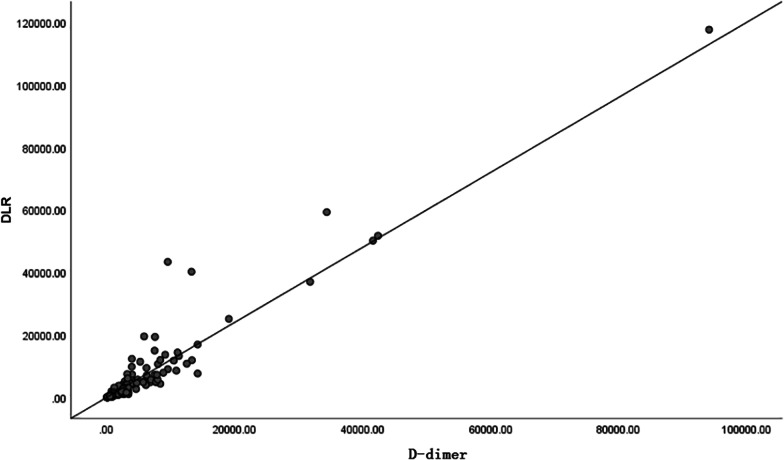
Scatterplot showing correlation between DLR and D-dimer for AAD patients

**Fig. 3:**
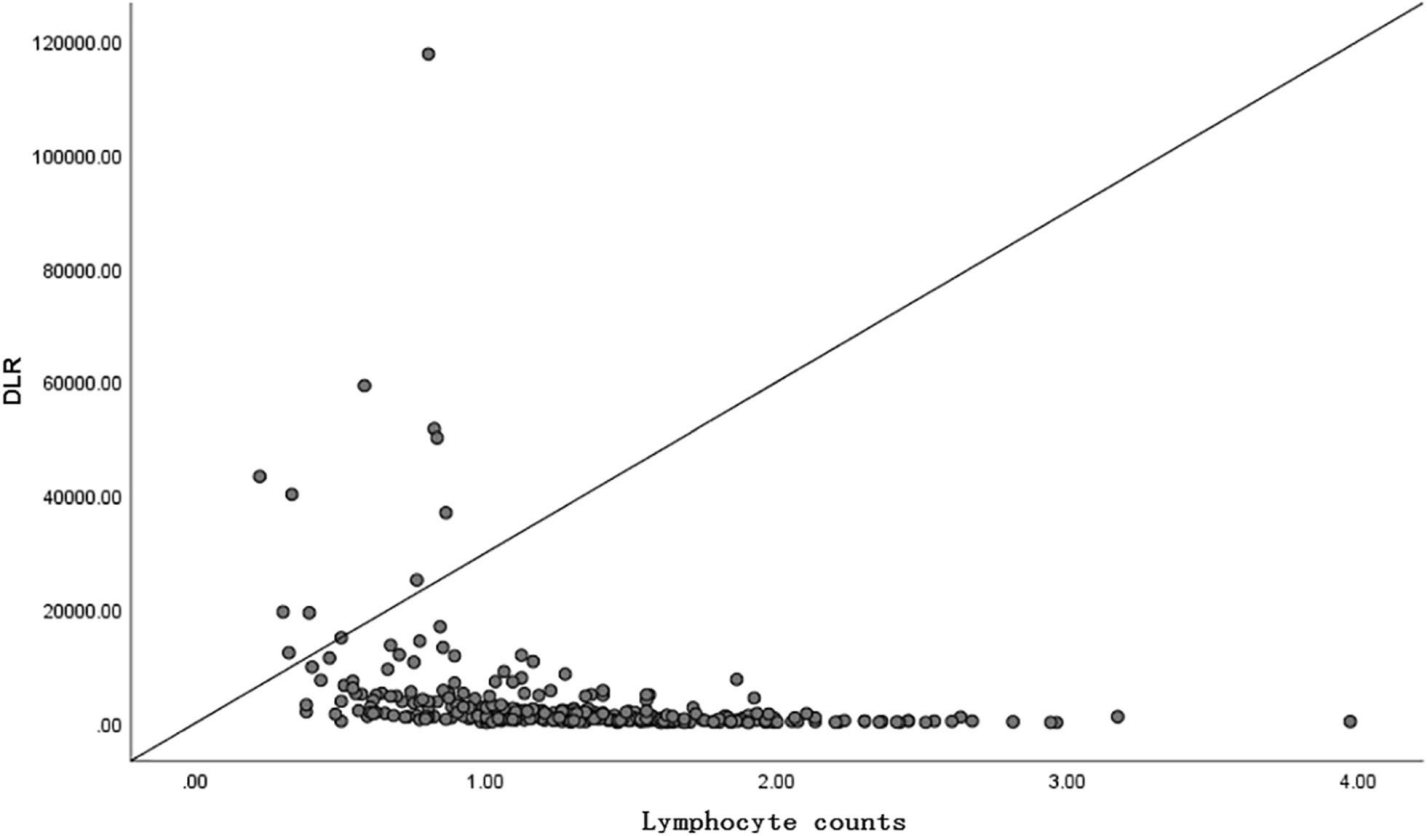
Scatterplot showing correlation between DLR and Lymphocyte counts for AAD patients

The present study demonstrated a positive correlation with DLR and D-dimer (*r* = 0.951, *p* = 0.000). A positive correlation (*r* = − 0.36, *p* = 0.000) between DLR and Lymphocyte counts was observed (Fig. 2, 3).

### The cutoff value, sensetivity, specificityof DLR

**Fig. 4:**
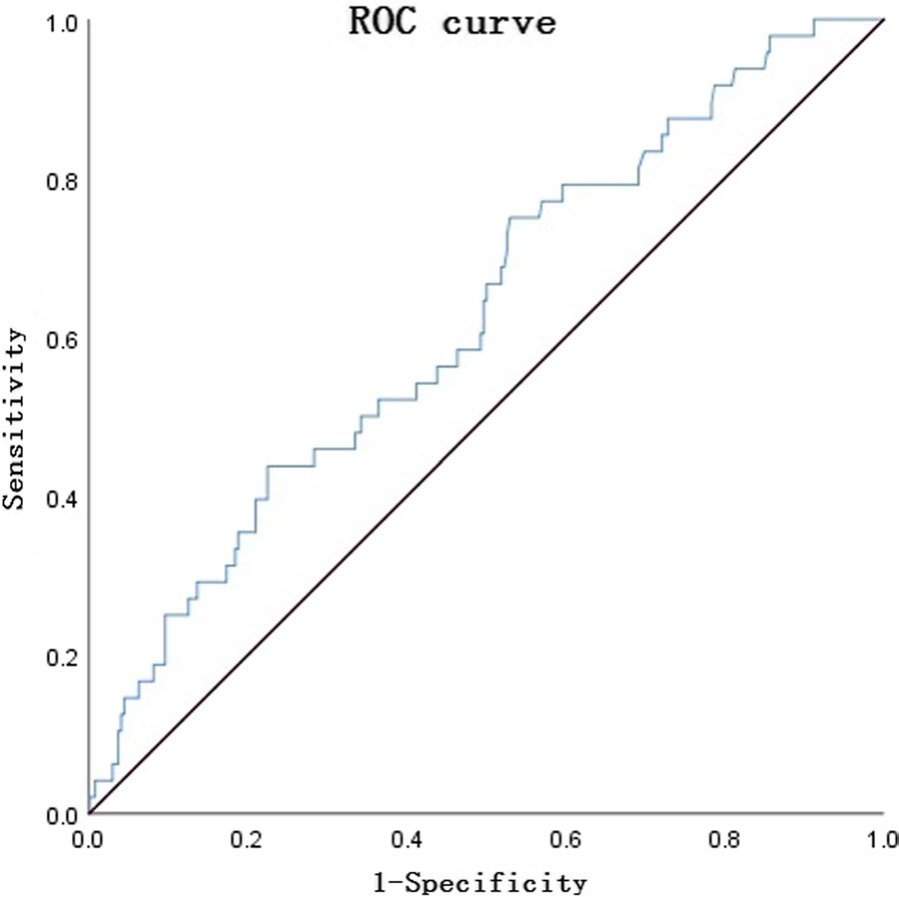
ROC curve of DLR

The survival receiver operating characteristic (ROC) curve was used for statistical analysis by IBM SPSS 26.0 software. The cut-off value of DLR for the diagnosis of in-hospital mortality should be set at < 907. ROC curve area is 0.625,and the sensitivity and specificity are 0.75 and 0.50 (Fig. 4).

### Association between DLR and in-hospital mortality Risk

The association between variables and in-hospital mortality risk is shown in Table 2. Univariate logistic regression analyses adjusted for covariates (age, sex) revealed that admission WBC levels (adjusted OR = 1.039; 95% CI = 1.016–1.175), neutrophil count (adjusted OR = 1.111; 95% CI = 1.031–1.198), lymphocyte count (adjusted OR = 0.495; 95% CI = 0.256–0.956), NLR (adjusted OR = 1.065; 95% CI = 1.013–1.119), PT (adjusted OR = 1.340; 95% CI = 1.118–1.606), HR (adjusted OR = 1.021; 95%CI = 1.008–1.035), and D-dimer (adjusted OR = 1.000; 95% CI = 1.000–1.000) had a significant impact on in-hospital mortality risk. The DLR (odds ratio [OR] 2.127, 95% CI 1.034–4.373, *P* = 0.040), HR (odds ratio [OR] 1.016, 95% CI 1.002–1.030, *P* = 0.029) and PT (odds ratio [OR] 1.231, 95% CI 1.018–1.189, *P* = 0.032) remained significant in multivariate analysis (Table 3).

**Table 2:** The correlation between DLR and parameters

Variables	DLR		F/t	*P*
	<907	≥ 907		
Sex male/female	115/25	158/22	1.996	0.158
Age, mean (SD)	51.4(11.8)	52.1(11.1)	0.129	0.543
Dissection type A/B	48/92	55/125	0.502	0.479
Hypertension No/Yes	50/90	50/130	2.309	0.129
Diabetes No/Yes	134/6	174/6	0.198	0.656
Smoking No/Yes	69/71	93/87	0.179	0.673
Drinking No/Yes	75/85	95/85	0.020	0.888
WBC, mean (SD)	9.8(3.3)	11.9(4.1)	2.932	**0.000**
HGB, mean (SD)	122.7(17.9)	118.6(22.2)	5.320	0.070
PLT, mean (SD)	254(98)	196(90)	1.221	**0.000**
NEUT, mean (SD)	6.9(3.0)	9.7(3.8)	4.281	**0.000**
LYM, mean (SD)	1.6(0.5)	1.0(0.3)	3.572	**0.000**
PLR, md (IQR)	141.3(111.1–208.4)	178.9(133.2–256.7)	7.620	**0.000**
NLR, md (IQR)	4.1(2.7–5.9)	9.6(6.6–13.6)	49.599	**0.000**
PT, mean (SD)	11.5(1.2)	12.3(1.7)	8.042	**0.000**
FIB, mean (SD)	5.0(3.7)	4.6(1.8)	2.257	**0.016**
APTT, mean (SD)	31.8(4)	31.5(5.6)	1.028	0.700
TC, mean (SD)	4.6(1.2)	4.2(1.0)	1.301	**0.012**
TG,, md (IQR)	1.2(0.9–1.7)	1.1(0.8–1.6)	2.360	0.159
HDL, mean (SD)	1.0(0.3)	1.0(0.3)	0.152	0.580
LDL, mean (SD)	2.7(1.0)	2.4(0.8)	1.932	**0.007**
CR, md (IQR)	88.5(67.5–114)	95.5(74.2–158.2)	23.845	**0.001**
UA,, mean (SD)	371(152)	390(183)	5.286	0.313
BNP,md (IQR)	832(120–932)	635(201–1730)	25.690	**0.000**
CK, md (IQR)	74.5(47–144)	89.5(55–238)	7.701	0.175
CKMB, md (IQR)	13(10–16)	14(10–18)	4.996	0.155
LDH, md (IQR)	250(223–356)	261(214–335)	11.948	**0.001**
HR,mean (SD)	86(18)	90(22)	1.950	0.087
SBP, mean (SD)	143(30)	144(31)	0.114	0.792
DBP,mean (SD)	86(18)	85(18)	0.766	0.915
D-dimer, md (IQR)	612(355–870)	2691(1795–4359)	31.033	**0.000**
BMI, mean (SD)	23(3.8)	22(4.0)	0.332	0.613

Bold value indicates *P* value < 0.05

**Table 3:** Univariate and Multivariate analyses of prognostic factors for in-hospital mortality in patients with AAD

Variables	Univariate analysis		Multivariate analysis	
	Adjusted OR,95% CI	*P*	OR, 95% CI	*P*
Dissection type	0.749(0.393–1.143)	0.381		
Hypertension	0.000(0.000–1.001)	0.797		
Diabetes	0.664(0.355–1.241)	0.999		
Smoking	0.664(0.355–1.241)	0.199		
Drinking	0.778(0.416–1.454)	0.432		
WBC	1.092(1.015–1.175)	**0.018**		
HGB	0.989(0.975–1.004)	0.139		
PLT	1.000(0.997–1.003)	0.832		
NEUT	1.111(1.030–1.198)	**0.006**		
LYM	0.051(0.261–0.963)	**0.038**		
PLR	1.002(0.999–1.004)	0.269		
NLR	1.063(1.012–1.117)	**0.015**		
PT	1.337(1.115–1.603)	**0.002**	1.231(1.018–1.489)	**0.032**
FIB	0.954(0.796–1.142)	0.606		
APTT	1.014(0.959–1.073)	0.615		
TC	0.903(0.678–1.204)	0.488		
TG	0.864(0.594–1.256)	0.443		
HDL	0.759(0.301–1.916)	0.559		
LDL	0.929(0.665–1.299)	0.668		
CR	1.001(1.000–1.002)	0.100		
UA	1.002(1.000–1.003)	0.080		
BNP	1.000(1.000–1.000)	**0.040**		
CK	1.000(1.000–1.000)	0.145		
CKMB	1.003(0.999–1.007)	0.152		
LDH	1.001(1.000–1.001)	0.084		
HR	1.021(1.007–1.035)	**0.003**	1.016(1.002–1.030)	**0.029**
SBP	0.995(0.985–1.005)	0.304		
DBP	0.994(0.978–1.010)	0.442		
D-dimer	1.000(1.000–1.000)	**0.025**		
BMI	0.950(0.876–1.030)	0.211		
DLR	2.576(1.281–5.184)	**0.008**	2.127(1.034–4.373)	0.040

Bold value indicates *P* value < 0.05

*Adjusted OR* Adjusted for age(< 52, ≥ 52), sex(male, female)

### Construction of a nomogram for in-hospital mortality risk

**Fig. 5:**
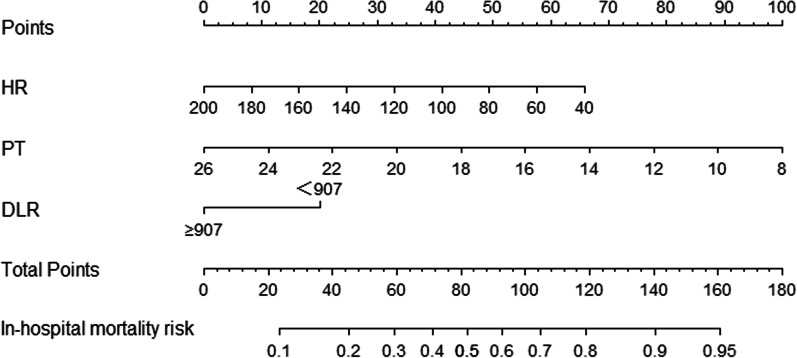
Nomogram of risk of in-hospital mortality

**Fig. 6:**
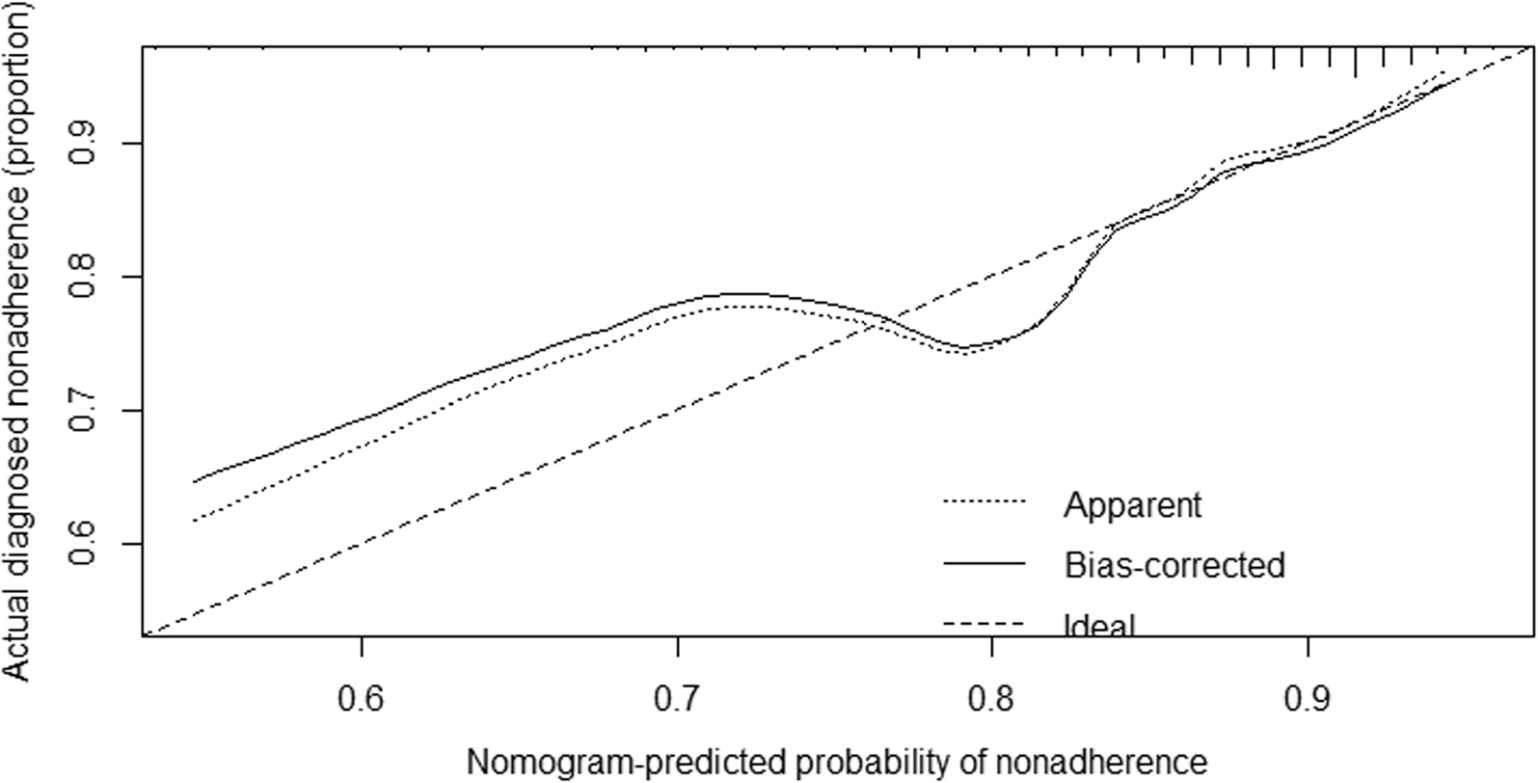
Prediction model fitting curve

Figure 5 shows a nomogram constructed by independent risk factors after multivariate analysis for in-hospital mortality risk. The nomogram’s Cindices for prediction of in-hospital mortality risk was 0.668. The calibration curves for in-hospital mortality risk exhibited high consistency between the values predicted by the nomogram and the actual observations (Fig. 6).

## Discussion

The purpose of this study was to investigate the prognostic relationship between DLR and AAD. To decrease the heterogeneity of research subjects, we focused the investigation on the early phase of the disease(within the first 48 h after symptom onset). AAD patients with high admission DLR levels is a powerful predictor of in-hospital mortality after adjusting for confounding factors in this retrospective study in addition to high HR and PT. In comparison to patients with a low level of DLR, patients with a value > 907 had a relatively high preoperative WBC and NEUT count, PLR, NLR, PT, CR, LDH, D-dimer, low preoperative platelet and LYM count, FIB, TC, LDL, and BNP. In addition, our results suggest that in-hospital and its importance increases progressively with higher HR and PT. To our knowledge, this is the first time that DLR has been investigated for its clinical utility in the evaluation of in-hospital mortality in patients with AAD.

A growing body of literature has shown that inflammatory cells, such as WBCs [21], CRP [22], neutrophils [23], platelets [24], and a combination of neutrophils/platelets and lymphocytes [16, 22], play an important role in the development and progression of aortic dissection. Among inflammatory indictors, immule infiltration of the aortic wall with lymphocytes has attracted increasing attention from researchers as an independent prognostic factor of AD-related diseases in recent years [25]. Most studies have demonstrated that inflammatory cells such as lymphocytes are able to induce apoptosis of smooth muscle cells and synthesis of metalloproteases [26, 27]. However, the exact mechanism by which lymphocytes influence the prognosis of arterial disease in detail,including AD, remains unclear. Studies have found that lymphopenia is associated with the progression of atherosclerosis [28], and a low level of lymphocytes in peripheral blood may be caused by lymphocyte apoptosis in atherosclerotic lesions, which gradually increases with atherosclerotic burden [29]. del Porto et al. showed that T lymphocytes were poorly represented in the aortic media, and a significant decrease in total T lymphocytes and T helper fractions was found in the peripheral blood of patients with AAD by flow cytometry [14]. Soon afterwards, Bedel et al. described that critical patients with lymphopenia, including AAD, had poor prognosis [16], which is consistent with the results presented here.

As a degradation product of cross-linked fibrin, D-dimer has become an important complementary tool in the diagnosis of thrombotic plasticity diseases and has also been used in the diagnosis and prognosis of acute AD. A negative D-dimer result may be useful to help rule out acute AD with a sensitivity of 100% [30]. Patients with D-dimer < 0.1 mg/mL will exclude AAD in all suspected cases [31]. Consistent results were observed in several studies in which elevated D-dimer levels were associated with early mortality and postoperative complications [32, 33]. Admission D-dimer levels (> 6.10 mg/ml) were associated with an increased risk of in-hospital death [19]. The D-dimer level in patients with AAD was influenced by dissection type. Based on types of AD, D-dimer concentration (≥ 20 mg/ml) can serve as a powerful indicator for increased in-hospital mortality in Stanford type A AD patients [10].

The present research did not find D-dimer or lymphocytes to be an independent prognostic factor on multivariate analysis. A significant positive association between DLR and D-dimer/lymphocytes count were observed. Their values in predicting in-hospital mortality risk for AAD might be better interpreted when they were considered as a whole. The development process of aortic dissection is rather complex. The aggregation of D-dimer leads to thrombosis and blockage of microvessels or organs in the whole body, leading to organ dysfunction. During the occurrence or development of aortic dissection, the patient's body secretes a large amount of glucocorticoids and other substances, resulting in immunosuppression of the body. This reaction may reduce the body resistance of aortic dissection patients, Thus, it is easy to have risks related to decreased immunity, such as lung inflammation. This response can induce sandwich related mortality. D-dimer/lymphocyte count is a mixed model, which can better reflect the complex process of the body under the action of these two factors.

### Limitations

This study, although, is the first to examine the relationship between DLR and prognosis. Some limitations existed in this study. First, this was a single-center, small sample observational study. The present results may be affected by the regional characteristics of patients in our hospital, and caution needs to be taken in spreading the application of research findings. Second, the study was designed to focus on in-hospital mortality. The long-term clinical outcomes and complications should be equally observed. Third, although we constructed the nomogram of the model, due to the lack of a sufficient sample size, we did not conduct external verification of the model, which will affect the promotion and application of the model. In addition, there is a lack of research on the mechanism by which DLR affects vascular disease. Hence, further multicenter studies will be conducted to verify the current findings. As we know, it is better to use survival analysis if the authors have the data on time-to-event. However, such data on time-to-event is not available, objective factors limit the surival analysis.

## Conclusion

Finally, a high admission DLR level might be a powerful predictor for increased in-hospital mortality in patients with AD. A larger prospective sample size is needed to verify the effect of this factor.

## Data Availability

The raw data supporting the conclusions of this article will be made available by the authors, without undue reservation.

## Abbreviations

DLR: D-dimer to lymphocyte count ratio
AAD: Acute aortic dissection
WBC: White blood cells
NLR: Neutrophil/lymphocyte
PT: Prothrombin time
HR: Heart rate
ROC: Receiver operating characteristic
